# Supplementary material for: Decentralized federated learning through proxy model sharing
Source: Nat Commun. 2023 May 22;14:2899. doi: 10.1038/s41467-023-38569-4 (PMC10203322; doi:10.1038/s41467-023-38569-4)
Supplement: Supplementary file 1 — Supplementary File [file 41467_2023_38569_MOESM1_ESM.pdf]

Supplementary Information

ProxyFL: Decentralized Federated Learning through Proxy Model Sharing

Shivam Kalra[1,2,3] Junfeng Wen[4] Jesse C. Cresswell[1]
Maksims Volkovs[1*] H.R. Tizhoosh[2,3,5*]

[1]Layer 6 AI, Toronto, ON, Canada
[2]Kimia Lab, University of Waterloo, ON, Canada
[3]Vector Institute, Toronto, ON, Canada
[4]Carleton University, School of Computer Science, Ottawa, ON, Canada
[5]Rhazes Lab, Dept. of AI & Informatics, Mayo Clinic, Rochester, MN, USA

*Corresponding authors: maks@layer6.ai, tizhoosh.hamid@mayo.edu

A Experiment Details

A.1 Benchmark Image Classification

We used the model architectures from Shen et al. (2020) for direct comparison. Specifically, the MLP consists of two hidden layers with 200 units each and ReLU activations. The CNN1 model consists of (conv(3×3, 6), ReLU, maxpool(2×2), conv(3×3, 16), ReLU, maxpool(2×2), fc(64), ReLU, fc(10)) where conv(3×3, 6) means a 3×3 convolution layer with 6 channels, maxpool(2×2) is a max pooling layer with 2×2 kernel, and fc(64) means a fully connected layer with 64 hidden units, etc. The CNN2 model is (conv(3×3, 128), ReLU, maxpool(2×2), conv(3×3, 128), ReLU, maxpool(2×2), fc(10)).

B Additional Results

B.1 Benchmark Image Classification

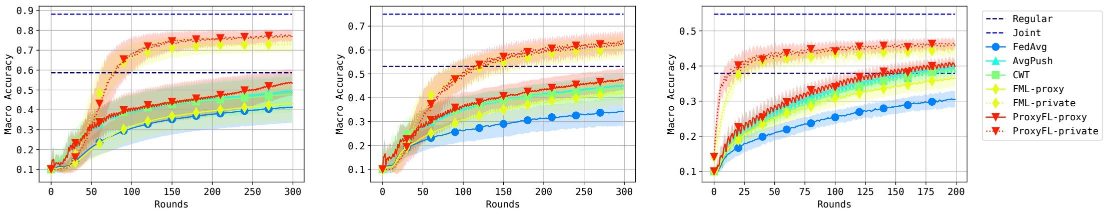

(a) MNIST (b) Fashion-MNIST (c) CIFAR10

Supplementary Figure 1: Macro-accuracy of the test performance with DP training, reporting means and standard devia-tions over clients and runs.

*Equal contribution.

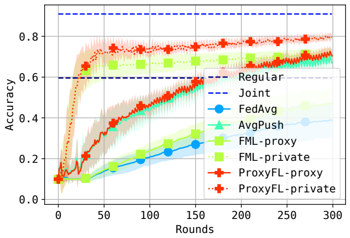

Supplementary Figure 2: MLP for both private and proxy.

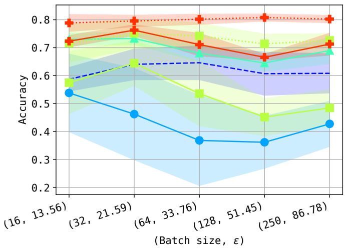

Supplementary Figure 3: Effect of batch size.

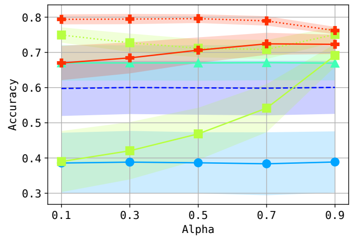

Supplementary Figure 4: Effect of DML weight.

In this section we provide additional experimental results for benchmarking on common image classification datasets. Fig. 1 shows the macro-accuracies, similar to the accuracies shown in the main text. Macro-accuracy is computed by first evaluating the accuracy per class then averaging the class accuracies. In settings with imbalanced data, it can be easy to achieve high accuracies without meaningful learning, whereas high macro-accuracy requires good performance on all classes. In our experiments classes are imbalanced with each client having a majority class. Nevertheless, we see that the macro-accuracies are very similar to the accuracies in the main text, showing that the methods are not biased towards the majority class.

Next we show additional ablation studies on MNIST. In the main text the private models for ProxyFL and FML were different from the proxy/centralized models. Fig. 2 shows the training curves when all models used the same MLP architecture. We see that ProxyFL-private can achieve the best performance regardless of the private model structure, as compared with the main text.

Increasing mini-batch size is beneficial for the stability of stochastic gradient updates and for computation speed. However, smaller batch sizes lead to stronger (ϵ, δ) privacy guarantees when using DP-SGD (Abadi et al., 2016). In the main text, for computation efficiency we used $B = 250$ images per DP-SGD step. Fig. 3 shows how batch size can affect the privacy guarantee. Smaller batch sizes do not have a significant effect on the final accuracy of ProxyFL-private, while the privacy guarantee dramatically improves. Therefore, we can have a strong privacy guarantee with ProxyFL by using small batch sizes at the cost of longer training time.

Finally, Fig. 4 shows the performance with different DML weights α, β. Note that this only affects ProxyFL and FML. We fix $\alpha = \beta$ in the experiments. We can see that ProxyFL is very robust to different choices of α, unlike its centralized counterpart FML. With a larger α, the performance gap between the private and proxy models gets smaller, which is reasonable as they tend to mimic each other.

Supplementary References

Martin Abadi, Andy Chu, Ian Goodfellow, H Brendan McMahan, Ilya Mironov, Kunal Talwar, and Li Zhang. Deep learning with differential privacy. In *Proceedings of the 2016 ACM SIGSAC conference on computer and communications security*, pages 308–318, 2016.

Tao Shen, Jie Zhang, Xinkang Jia, Fengda Zhang, Gang Huang, Pan Zhou, Kun Kuang, Fei Wu, and Chao Wu. Federated mutual learning. *arXiv preprint arXiv:2006.16765*, 2020.
